# Model Predicting Survival in Intermediate-Stage HCC Patients Reclassified for TACE Based on the 2022 BCLC Criteria

**DOI:** 10.3390/cancers17050894

**Published:** 2025-03-05

**Authors:** Jihoon Kim, Jin-Hyoung Kim, Eunbyul Ko, Jeong-Yeon Kim, Byung Soo Im, Gun Ha Kim, Hee Ho Chu, Heung-Kyu Ko, Dong Il Gwon, Ji Hoon Shin, Ibrahim Alrashidi

**Affiliations:** 1 Department of Radiology, Research Institute of Radiology, College of Medicine, Asan Medical Center, University of Ulsan, Seoul 05505, Republic of Korea; jihoonkim@amc.seoul.kr (J.K.); byeouly@amc.seoul.kr (E.K.); n151114@amc.soeul.kr (J.-Y.K.); kofi@amc.seoul.kr (B.S.I.); kimgh.rad@amc.seoul.kr (G.H.K.); d180717@amc.seoul.kr (H.H.C.); hk.ko@amc.seoul.kr (H.-K.K.); radgwon@amc.seoul.kr (D.I.G.); jhshin@amc.seoul.kr (J.H.S.); 2 Department of Radiology, Prince Sultan Military Hospital, Madinah 42375, Saudi Arabia; ibaalrashidi@moh.gov.sa

**Keywords:** liver, hepatocellular carcinoma, transarterial chemoembolization, prognostic model, overall survival

## Abstract

**Background/Objectives**: The Barcelona Clinic Liver Cancer (BCLC) staging system for hepatocellular carcinoma (HCC) was updated in 2022 to refine patient stratification, particularly in patients with intermediate-stage (BCLC B) HCC. Although transarterial chemoembolization (TACE) remains a key treatment for these patients, there is no prognostic model for survival outcomes based on the pretreatment factors of patients who meet the updated 2022 BCLC indications for TACE. The aim of this study was to develop a pretreatment risk model predicting overall survival (OS) in patients with intermediate-stage HCC and reclassified as candidates for TACE according to the updated 2022 BCLC criteria. **Methods**: This retrospective study included 658 HCC patients treated with first-line TACE according to the updated BCLC 2022 guidelines. Pretreatment factors such as the Child–Pugh score, tumor burden (up-to-11 criteria), bilobar tumor involvement, and serum alpha-fetoprotein (AFP) levels were analyzed. Cox proportional hazards models were used to identify significant predictors of OS, with these factors subsequently incorporated into a risk prediction model. **Results**: Significant predictors of OS included Child–Pugh score ≥ 7, bilobar tumor involvement, beyond up-to-11 criteria, and AFP ≥ 400 ng/mL. A risk model was developed using these factors, stratifying patients into low-, intermediate-, and high-risk groups. The median OS in the low-, intermediate-, and high-risk groups was 53, 35, and 21 months, respectively. **Conclusions**: The proposed pretreatment risk prediction model may be useful for predicting OS and guiding TACE candidacy in intermediate-stage HCC patients based on the updated 2022 BCLC guidelines.

## Simple Summary

Hepatocellular carcinoma (HCC) is a common type of liver cancer, and for patients with intermediate-stage disease, transarterial chemoembolization (TACE) is a widely used treatment. In 2022, the Barcelona Clinic Liver Cancer (BCLC) staging system was updated to improve treatment selection, but no model has been established to predict the survival outcomes for patients undergoing TACE under these new criteria. This study aims to develop a simple, practical model to help predict survival based on key pretreatment factors, such as liver function, tumor burden, and blood biomarkers. By analyzing data from 658 patients, researchers identified risk factors that can classify patients into low-, intermediate-, and high-risk groups. The findings suggest that this model can assist doctors in making more informed decisions about TACE candidacy, leading to better treatment strategies, and potentially improving the survival outcomes for patients with intermediate-stage HCC.

## 1. Introduction

Hepatocellular carcinoma (HCC) is one of the leading causes of cancer-related mortality worldwide. The management of HCC remains complex due to tumor heterogeneity and variations in the liver function reserve of patients. The Barcelona Clinic Liver Cancer (BCLC) staging system for hepatocellular carcinoma (HCC), first introduced in 1999, has been widely adopted as the standard framework for guiding HCC treatment decisions. It provides tailored recommendations based on the stage of the disease, and to further enhance its precision, the BCLC strategy was updated in 2022 to introduce a more refined classification system.

Transarterial chemoembolization (TACE) is mainly indicated for patients with intermediate-stage (BCLC stage B) HCC, particularly those with preserved liver function. Despite the established role of TACE in prolonging survival and achieving local tumor control [1,2,3,4], a patient’s response to treatment varies significantly depending on their tumor burden, liver function, and vascular anatomy, enhancing the complexity of managing this condition and necessitating a more refined selection process to optimize treatment outcomes. According to the revised 2022 BCLC strategy, TACE is indicated only for patients with a defined tumor burden that is accessible to selective catheterization and preserved liver function.

Prognostic models have been designed to predict overall survival (OS) in patients with HCC who undergo TACE [5,6,7,8]. To date, however, no prognostic model based on routinely available pretreatment parameters has been developed for HCC patients with revised BCLC B stage who are indicated for TACE. Furthermore, the emergence of newer systemic chemotherapeutic agents and of TACE combined with systemic therapy have expanded treatment indications leading to improved OS in patients with HCC. It is therefore essential to develop a precise prognostic model that can predict patient’s response to TACE and enable the selection of patients who will benefit the most from TACE. This model may also be useful in identifying patients unsuitable for TACE. The aim of this study was to evaluate the pretreatment factors predicting OS in patients with intermediate-stage HCC and reclassified as candidates for TACE according to the updated 2022 BCLC criteria. This study also aimed to develop a prognostic model based on these factors in order to identify patients who are more likely to benefit from TACE while suggesting alternative therapeutic strategies for those with a poor prognosis. Ultimately, this model supports clinicians in making evidence-based treatment decisions, improving personalized treatment strategies, and enhancing overall patient outcomes.

## 2. Materials and Methods

### 2.1. Study Patients

**Figure 1:**
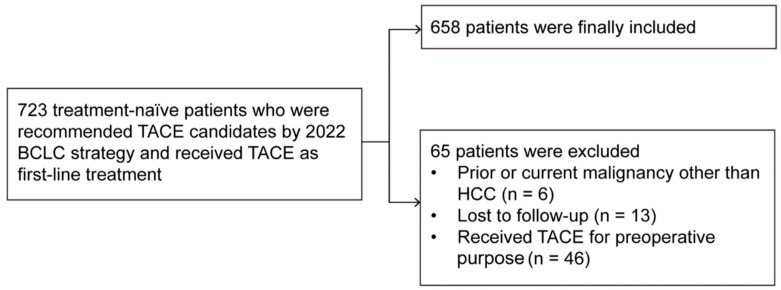
Patient selection flow chart.

This study was approved by the institutional review board of our institution, which waived the requirement for informed consent because of the retrospective design of the study. HCC was diagnosed according to the guidelines of the American Association for the Study of Liver Diseases (AASLD) or the European Association for the Study of the Liver (EASL) [9,10] and was staged according to the BCLC staging system. Patients with intermediate-stage HCC were included if they were candidates for TACE according to the 2022 updated BCLC strategy, were naive to previous treatment, and had undergone TACE as a first-line treatment. Patients were excluded if they had a prior or current malignancy other than HCC, if they had been lost to follow-up, or if they had undergone TACE for preoperative purposes (Figure 1).

### 2.2. TACE

The details of the TACE procedure have been described previously [11]. TACE was performed by one of eight experienced interventional radiologists, each with at least 10 years of experience with TACE. Cisplatin-based chemoembolization (2 mg/kg body weight) was performed using a 1.7–2.4-F microcatheter (Progreat Lambda, Terumo, Tyokyo, Japan; Renegade, Boston Scientific, Cork, Ireland; Carnelian, Tokai Medical Products, Aichi, Japan). A 1:1 emulsion of cisplatin and iodized oil (Lipiodol^®^, Guerbet, Paris, France) was infused into the feeding artery; this was followed by embolization with gelatin particles (Upjohn, Kalamazoo, MI, USA) until arterial flow stasis was observed.

### 2.3. Follow Up

The patients were initially followed up 1 month after TACE with laboratory tests and contrast-enhanced computed tomography (CT) or magnetic resonance imaging. Patients were subsequently followed up every 2–3 months for the first 2 years, and every 3–6 months thereafter until the recurrence of HCC. TACE was repeated in patients who showed an insufficient response after a single session and in those with recurrent tumors.

### 2.4. Data Analysis

The primary study endpoints were OS and the development of a pretreatment risk model that was able to predict OS. The pretreatment factors that were tested included the patient’s age, sex, HCC etiology, Child–Pugh score (5–6 vs. 7), within or beyond the up-to-11 criteria, the extent of tumor involvement (unilobar vs. bilobar), and the serum alpha-fetoprotein (AFP) concentration (<400 ng/mL vs. ≥400 ng/mL). The up-to-11 criteria combined the number of tumors and the size of the largest tumor, with the sum being no more than 11 [12,13].

To generate a pretreatment risk prediction model for OS, univariable and multivariable Cox proportional hazards analyses were performed to identify the pretreatment factors significantly associated with OS. Variables showing *p*-values < 0.05 in the univariate analyses were included in a multivariable analysis model using the backward elimination method. Risk points were then assigned to these variables according to their β regression coefficients, and the prediction model was developed [14]. Patients were divided into three prognostic categories (low, intermediate, and high-risk groups) based on changes in the risk estimates for each point increase in scores. The OS outcomes were evaluated using the Kaplan–Meier method and compared using log-rank tests.

The secondary study endpoints included the patient’s radiologic tumor response, progression-free survival (PFS), and major complications following TACE. Tumor response was evaluated 1 month following TACE using mRECIST criteria, with responses categorized as complete response (CR), partial response (PR), stable disease (SD), and progressive disease (PD) [15]. Patients who achieved CR or PR were classified as showing an objective tumor response. The rates of objective tumor response were compared using χ^2^ tests. PFS was defined as the time from the first TACE session until tumor progression, based on the mRECIST guidelines, or death from any cause. According to the Society of Interventional Radiology (SIR) reporting standards [16], major complications were defined as those necessitating additional treatment, including a hospital stay beyond the normal postoperative course, an increased level of care, substantial morbidity, or death (SIR classifications C–E). All statistical analyses were performed using SPSS software (version 21.0, SPSS, Chicago, IL, USA), with two-sided *p*-values < 0.05 considered statistically significant.

## 3. Results

### 3.1. Patient Characteristics

The study cohort consisted of 658 patients who were diagnosed with HCC and underwent TACE between January 2010 and June 2023. Of these patients, 89% were men, 67% were positive for hepatitis B virus (67%), 92% had Child–Pugh class A (score 5–6) liver function, 64% had bilobar tumor involvement, 72% had serum AFP concentrations < 400 ng/mL, and 55% were within the up-to-11 criteria (Table 1). The median maximal tumor size was 6 cm.

### 3.2. Overall Survival After TACE

During the follow-up period after TACE, 434 (66%) of the 658 patients had died and 224 (34%) remained alive. The 1-, 3-, 5-, and 10-year OS rates were 88%, 52%, 32%, and 15%, respectively, and the median OS after TACE was 37 months (95% confidence interval [CI], 34–40 months).

The multivariable Cox regression analyses found that Child–Pugh score of 7 (hazard ratio [HR], 1.60; *p* = 0.004), beyond up-to-11 criteria (HR, 1.54; *p* < 0.001), bilobar tumors (HR, 1.25; *p* = 0.039), and serum AFP concentration ≥ 400 ng/mL were significant risk factors for OS (Table 2).

These four risk factors were incorporated into a pretreatment risk prediction model. Risk points were assigned according to the β regression coefficients of these four risk factors, with two points each assigned for a Child–Pugh score of 7, beyond up-to-11 criteria, and serum AFP concentration ≥400 ng/mL; and one point assigned for bilateral tumors (Table 2 and Table 3).

**Figure 2:**
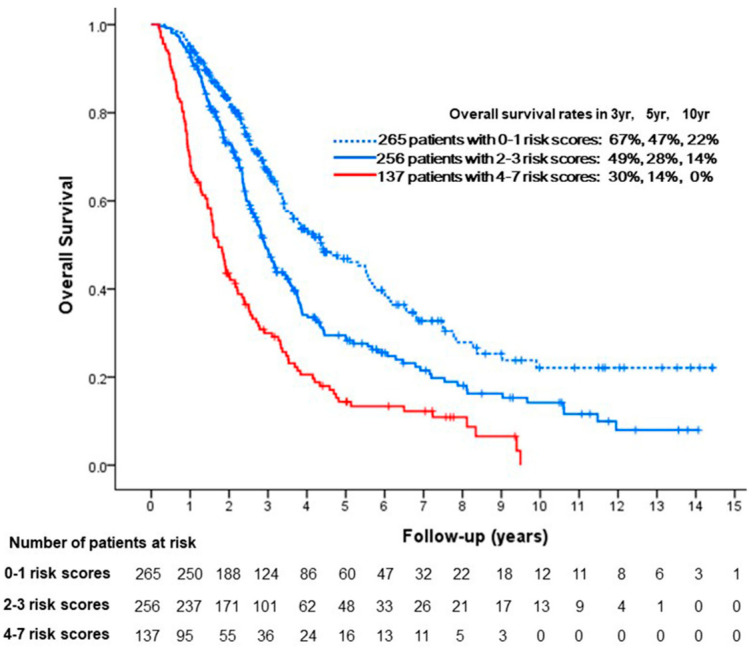
Kaplan–Meier analyses of overall survival in stratified risk groups for the entire study population.

The risk scores for all patients were calculated as the sum of these corresponding risk points, patients with scores of 0–1 (n = 265), 2–3 (n = 256), and 4–7 (n = 137) were categorized into the low-(A), intermediate-(B), and high-risk (C) subgroups, respectively. These three subgroups had a median OS of 53 months (95% CI, 39–66 months), 35 months (95% CI, 32–38 months), and 21 months (95% CI, 17–24 months), respectively (Figure 2).

**Figure 3:**
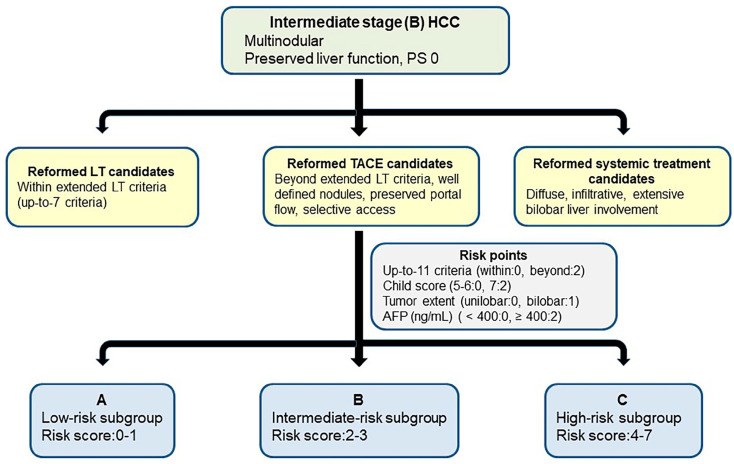
The proposed risk prediction model and subclassification of patients with intermediate-stage HCC who were newly reclassified as candidates for TACE according to the 2022 updated BCLC strategy. (HCC, hepatocellular carcinoma; LT, liver transplantation; TACE, transarterial chemoembolization).

**Figure 4:**
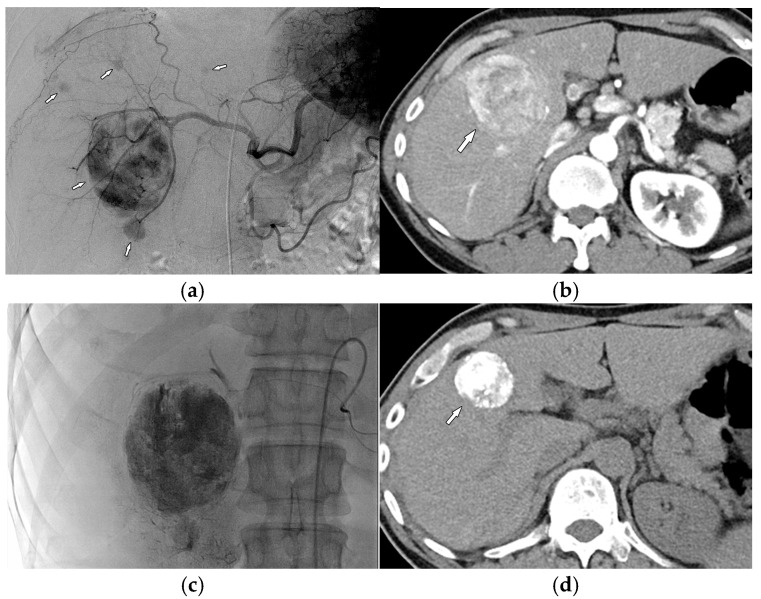
A 53-year-old man with BCLC B HCC identified as a candidate for TACE according to the 2022 updated BCLC strategy. (**a**) Common hepatic angiography image showing five HCCs (arrows) in bilobar locations; (**b**) axial CT image obtained in the arterial phase showing the largest HCC (diameter, 5.9 cm; arrow). The prediction model described in the present study classified this patient into the low-risk subgroup (risk score: 1); (**c**) performance of TACE; (**d**) non-enhanced CT image 4 years after initial TACE showing lipiodol uptake and a reduction in tumor size without recurrence (diameter, 3.5 cm; arrow).

The OS rates differed significantly between the A and B (*p* < 0.001) subgroups and between the B and C (*p* < 0.001) subgroups. The proposed risk prediction model and the subclassification of newly reclassified candidates for TACE are shown in Figure 3, with a representative patient described in Figure 4.

### 3.3. Radiologic Tumor Response, Progression-Free Survival, and Major Complications

Of the 658 patients, 195 (29%) achieved CR, 360 (55%) achieved PR, 75 (12%) had SD, and 28 (4%) showed PD 1 month after TACE. The overall objective tumor response rate was 84% (554/658), with these rates in the A, B, and C subgroups being 91% (240/265), 85% (219/256), and 70% (96/137), respectively (*p* < 0.001).

During follow-up, 557 (85%) patients showed tumor progression or died. The overall median PFS after TACE was 12 months (95% CI, 11–13 months), with the median PFS in the A, B, and C subgroups being 15, 11, and 7 months, respectively (*p* < 0.001).

Of the 658 patients, 34 (5%) experienced major complications, including allergic reactions related to cisplatin in eleven patients; acute renal failure and liver abscess in six patients; persistent fever in three patients; biloma and chemoembolization-related cholecystitis in two patients; and hepatic failure, sepsis, ileus, and liver infarction in one patient each.

## 4. Discussion

To our knowledge, this is the first study to evaluate the survival outcomes and safety of TACE as first-line treatment in patients with BCLC stage B HCC, based on the revised criteria for TACE introduced in the 2022 BCLC update. The large sample size (n = 658) enabled the development of a predictive model based on pretreatment factors. This model incorporated key prognostic factors, including the up-to-11 criteria, Child–Pugh score, bilobar tumor location, and serum AFP concentrations. Based on these factors, patients were classified into low-, intermediate-, and high-risk groups, with median OS of 53, 35, and 21 months, respectively.

The 2022 update of the BCLC classification system emphasized the importance of patients’ serum AFP levels, liver function, and tumor burden in determining the treatment administered to patients with HCC [17]. AFP may serve as a surrogate marker for tumor burden or aggressiveness and has been frequently identified as a significant predictor of survival in HCC patients [13,18,19]. Although recent advances in TACE techniques and the use of cone-beam CT have reduced the rates of liver function deterioration and reduced patients’ Child–Pugh score reductions [20,21,22], TACE remains linked to these complications [23,24]. TACE has also been associated with the further deterioration of liver function in patients who are already in a vulnerable condition, potentially resulting in devastating outcomes. Consequently, it is crucial to incorporate measures of liver function into prediction models for TACE. The results of the present study highlight the clinical importance of integrating factors that represent tumor burden and liver function into prognostic models for HCC patients undergoing TACE.

Several criteria for assessing tumor burden have been proposed [8,12,25,26,27], including the subclassification of patients with BCLC B stage HCC according to their tumor burden (up-to-7 criteria) [25]. This model, however, was originally developed for liver transplantation candidates, limiting the ability of these criteria to subclassify intermediate-stage HCC treated with TACE. Moreover, the up-to-7 criteria showed limited predictive performance in HCC patients treated with TACE [28,29]. A recently proposed subclassification system [12] that uses the up-to-11 criteria to define tumor burden has been validated in multiple cohorts [16,17,18,19]. These TACE criteria are preferred by some researchers when aiming to distinguish between a major and minor tumor burden in patients with HCC [13,30,31,32,33,34]. The present study indicated that the up-to-11 criteria were predictive of OS, suggesting that these criteria can improve treatment decisions for intermediate-stage HCC patients who are candidates for TACE according to the revised 2022 BCLC strategy.

The prediction model developed in this study showed that patients in the low-risk or A subgroup benefited the most from TACE treatment (median OS 53 months). The survival outcomes for patients in the intermediate-risk or B subgroup were less favorable, with a median OS of 35 months. Based on the 2022 BCLC strategy, BCLC stage B patients who are candidates for TACE are expected to have a median OS longer than 30 months [17]. Generally, patients are considered ideal candidates for TACE if their expected median OS exceeds this threshold [35,36]. Patients in the intermediate-risk subgroup in the present study were therefore regarded as ideal candidates for TACE. Taken together, these findings suggest that patients with intermediate-stage (BCLC stage B) HCC who are at a low and intermediate risk may be good candidates for first-line TACE.

Patients in the high-risk or C subgroup in the present study had a median OS of 21 months, suggesting that they may not be appropriate candidates for first-line TACE treatment. Their relatively poor survival outcomes suggest that alternative treatments, such as systemic therapy (atezolizumab plus bevacizumab) or the combinations of TACE with systemic therapy, may be more effective [32,37,38]. Patient stratification in our prediction model may enable personalized treatment planning [39], potentially guiding decisions regarding the administration of TACE and the need for adjunctive or alternative therapies.

Recent advancements in loco-regional therapies for intermediate-stage HCC have broadened treatment options beyond conventional TACE. A systematic review and meta-analysis comparing TACE and TARE demonstrated the potential advantages of TARE, particularly in selected patients [40]. However, despite these benefits, TARE has not yet replaced TACE as the standard of care due to its cost, availability, and the need for specialized expertise. The updated 2022 BCLC guidelines underscore the importance of a more personalized treatment strategy, emphasizing the need for future research to optimize the patient selection criteria for loco-regional therapies and explore novel combinations that could improve survival outcomes. However, there is a lack of research providing direct comparisons within the updated 2022 BCLC framework, highlighting the need for further research on the optimization of treatment sequencing and exploring the comparative effectiveness of TACE versus TARE for clinical decision-making.

This study had several limitations. First, although the data were derived from a relatively large patient cohort, the retrospective design and single-center setting of this study may limit the generalizability of its findings, suggesting the need for future prospective external validation studies. Second, the study cohort was relatively homogeneous racially and ethnically, as all included patients were from South Korea. Thus, the present results may be inapplicable to HCC patients in other countries, particularly in Western nations, due to variations in demographic characteristics and causes of liver diseases. Third, this study did not include data regarding post-progression survival (PPS) or detailed information on post-progression treatments. As the study was retrospectively designed to assess the prognostic factors associated with TACE candidacy, the systematic collection of PPS data was beyond its scope. However, post-progression outcomes play a crucial role in guiding treatment strategies and overall patient management. Future studies should incorporate PPS and post-progression treatment data to further refine the risk stratification and provide a more comprehensive understanding of the long-term outcomes of patients undergoing TACE. Additionally, this study is limited by the absence of external validation for the proposed prognostic model, leaving its generalizability to other populations and clinical settings unconfirmed. Prospective multicenter studies are needed to externally validate the model and ensure its applicability and robustness across diverse patient cohorts.

## 5. Conclusions

The prediction model described in this study may serve as a practical and reliable tool for identifying patients with intermediate-stage HCC who are candidates for TACE based on the 2022 updated BCLC strategy. Patients in the low-risk or A subgroup, characterized by tumor burdens within the up-to-11 criteria, a Child–Pugh score of 5–6, a unilobar tumor location, and serum AFP levels < 400 ng/mL, will likely derive the greatest benefit from TACE. The proposed model provides a valuable tool for personalized risk stratification in intermediate-stage HCC patients, potentially enhancing clinical decision-making and improving patient outcomes. However, the study is limited by the absence of external validation and the relatively small sample size, which only included patients from a single center. Future prospective multicenter studies are needed to validate the model across diverse populations and enhance its generalizability.

## Figures and Tables

**Table 1:** Baseline Demographic and Clinical Characteristics of the Patients Included in this Study.

Variable	All Patients
Patients	658
Age, year, mean ± SD (range)	60.8 ± 10.2 (27–89)
Sex, n (%)	
Male	588 (89)
Female	70 (11)
Etiology of HCC, n (%)	
HBV	444 (67)
HCV	63 (10)
Alcoholics	79 (12)
Others	72 (11)
Child–Pugh score, n (%)	
5–6	604 (92)
7	54 (8)
Up-to-11 criteria, n (%)	
Within	359 (55)
Beyond	299 (45)
Tumor location, n (%)	
Unilobar	237 (36)
Bilobar	421 (64)
AFP (ng/mL), n (%)	
≥400	184 (28)
<400	474 (72)

Values in parentheses indicate the percentage of the total number of patients in each category. SD, standard deviation; HBV, hepatitis B virus; HCV, hepatitis C virus; AFP, alpha-fetoprotein.

**Table 2:** Univariable and multivariable analyses of factors associated with overall survival after TACE.

	Univariable Analysis			Multivariable Analysis				
Variables	HR	95% CI	*p*-Value	HR	95% CI	*p*-Value	Beta Coefficients	Risk Point
Age > 60 years	1.18	0.98–1.43	0.08	-	-	-		
Male sex	1.04	0.71–1.31	0.82	-	-	-		
Etiology								
HBV	1		0.26	-		-		
HCV	1.32	0.62–2.24	0.07	-	-	-		
Alcoholic	1.10	0.45–2.52	0.53	-	-	-		
Others	1.19	0.46–2.15	0.30	-	-	-		
Child–Pugh score 7	1.81	1.32–2.50	<0.001	1.60	1.16–2.21	0.004	0.47	2
Beyond up-to-11 criteria	1.75	1.45–2.11	<0.001	1.54	1.26–1.88	<0.001	0.43	2
Bilobar tumors	1.43	1.17–1.75	0.001	1.25	1.01–1.54	0.039	0.22	1
Serum AFP level ≥ 400 ng/mL	1.70	1.39–2.07	<0.001	1.56	1.27–1.90	<0.001	0.44	2

HR, hazard ratio; CI, confidence interval; HBV, hepatitis B virus; HCV, hepatitis C virus; AFP, alpha-fetoprotein.

**Table 3:** The proposed model’s prediction of the overall survival of TACE candidates with BCLC-B HCC reclassified according to the updated 2022 criteria.

Points	0	1	2
Child score	5–6		7
Up-to-11	In		Out
Tumor extent	Unilobar	bilobar	
AFP (ng/mL)	<400		≥400
**Risk score**	**Class**		
0–1	A		
2–3	B		
4–7	C		

## Data Availability

The data presented in this study are available upon justified request.

## Abbreviations

HCC	hepatocellular carcinoma
BCLC	Barcelona Clinic Liver Cancer
TACE	transarterial chemoembolization
OS	overall survival
AFP	alpha-fetoprotein
AASLD	American Association for the Study of Liver Diseases
EASL	European Association for the Study of the Liver
CT	computed tomography
PFS	progression-free survival
CR	complete response
PR	partial response
SD	stable disease
PD	progressive disease
SIR	Society of Interventional Radiology
